# Assessing climate niche similarity between persian fallow deer (*Dama mesopotamica*) areas in Iran

**DOI:** 10.1186/s12862-024-02281-8

**Published:** 2024-07-05

**Authors:** Ehsan Rahimi, Pinliang Dong, Faraham Ahmadzadeh

**Affiliations:** 1 Environmental Sciences Research Institute, Shahid Beheshti University, Tehran, Iran; 2 Department of Geography and the Environment, University of North Texas, Fort Worth metroplex, Dallas, USA

**Keywords:** Persian fallow deer, *Dama mesopotamica* ecological niche modeling, Niche divergence, Extrapolation analysis

## Abstract

The Persian fallow deer or Mesopotamian fallow Deer (*Dama mesopotamica*, Brook 1875), a species of significant ecological importance, had faced the threat of extinction in Iran. One conservation strategy involved the translocation of Persian deer to enclosed areas across Iran, where they were afforded protection from external threats and provided with essential care by human caretakers. While human caretakers diligently attend to their needs and mitigate external threats, climate variables may now become critical factors affecting population dynamics in enclosed areas. This study aims to assess the similarity in climate niches between the original area (Dez and Karkheh) of the Persian deer species and 11 newly enclosed areas. To achieve this, we employed climate data and ecological niche modeling (ENM) techniques to assess the variations in climate among 12 areas. We utilized the environmental equivalency test to determine whether the environmental spaces of area pairs exhibit significant differences and whether these spaces are interchangeable. Extrapolation analyses were also constructed in the next steps to explore climatic conditions in original fallow deer habitats that are non-analogous to those in other parts of Iran. Our results reveal significant disparities in climate conditions between the original and all translocated areas. Based on observations of population growth in specific enclosed areas where translocated deer populations have thrived, we hypothesize that the species may demonstrate a non-equilibrium distribution in Iran. Consequently, these new areas could potentially be regarded as part of the species’ potential climate niche. Extrapolation analysis showed that for a significant portion of Iran, extrapolation predictions are highly uncertain and potentially unreliable for the translocation of Persian fallow deer. However, the primary objective of translocation efforts remains the establishment of self-sustaining populations of Persian deer capable of thriving in natural areas beyond enclosed areas, thus ensuring their long-term survival and contributing to preservation efforts. Evaluating the success of newly translocated species requires additional time, with varying levels of success observed. In cases where the growth rate of the species in certain enclosed areas falls below expectations, it is prudent to consider climate variables that may contribute to population declines. Furthermore, for future translocations, we recommend selecting areas with climate similarities to regions where the species has demonstrated growth rates.

## Introduction

### Fundamental niche vs. realized niche

The concept of “niche” encompasses the ecological conditions necessary for a species to maintain populations in a specific area, including its requirements and impacts on resources, other species, area, and environment [1]. Initially, Grinnell defined niche as the range of environmental conditions supporting a species’ growth and success, primarily considering broad-scale species distributions [2, 3]. However, at smaller scales, incorporating models of resource utilization, and consequences, Charles Elton introduced the concept of “Eltonian niche,” focusing on local interactions [4]. “Grinnellian niche” is viewed as characteristics evaluated through scenopoetic variables (which are not consumed by species), influenced by heritable factors, across the species’ geographic range. In contrast, the “Eltonian niche” describes interactions between a species and its immediate resources and other organisms [1, 3].

Hutchinson later defined the ecological niche as a hypervolume shaped by the environmental conditions allowing a species to ‘exist indefinitely’ [5]. This concept inspired a new generation of ecologists and biogeographers to create ecological niche hypervolumes using species occurrence data and environmental variables, known as “ecological niche” or “species distribution” models (SDMs) [6]. In Hutchinson’s framework, the “fundamental niche” comprised all environmental states capable of sustaining a species, reflecting its physiology and behavior, irrespective of specific locations. In contrast, the “realized niche” denotes the subset of the fundamental niche where a species outcompetes others and thrives. Niches can be contrasted either in geographic space (G-space) or environmental space (E-space). G-space refers to comparing the distribution of multiple species within the same geographic area, while E-space involves comparing environmental conditions like climate, soil type, vegetation, or other ecological factors within the same geographic area or across different geographic areas (e.g., native and non-native regions) for a single species. Utilizing E-space allows for a more comprehensive evaluation of niche overlap as it considers environmental availability and similarity between ranges [7].

**Fig. 1:**
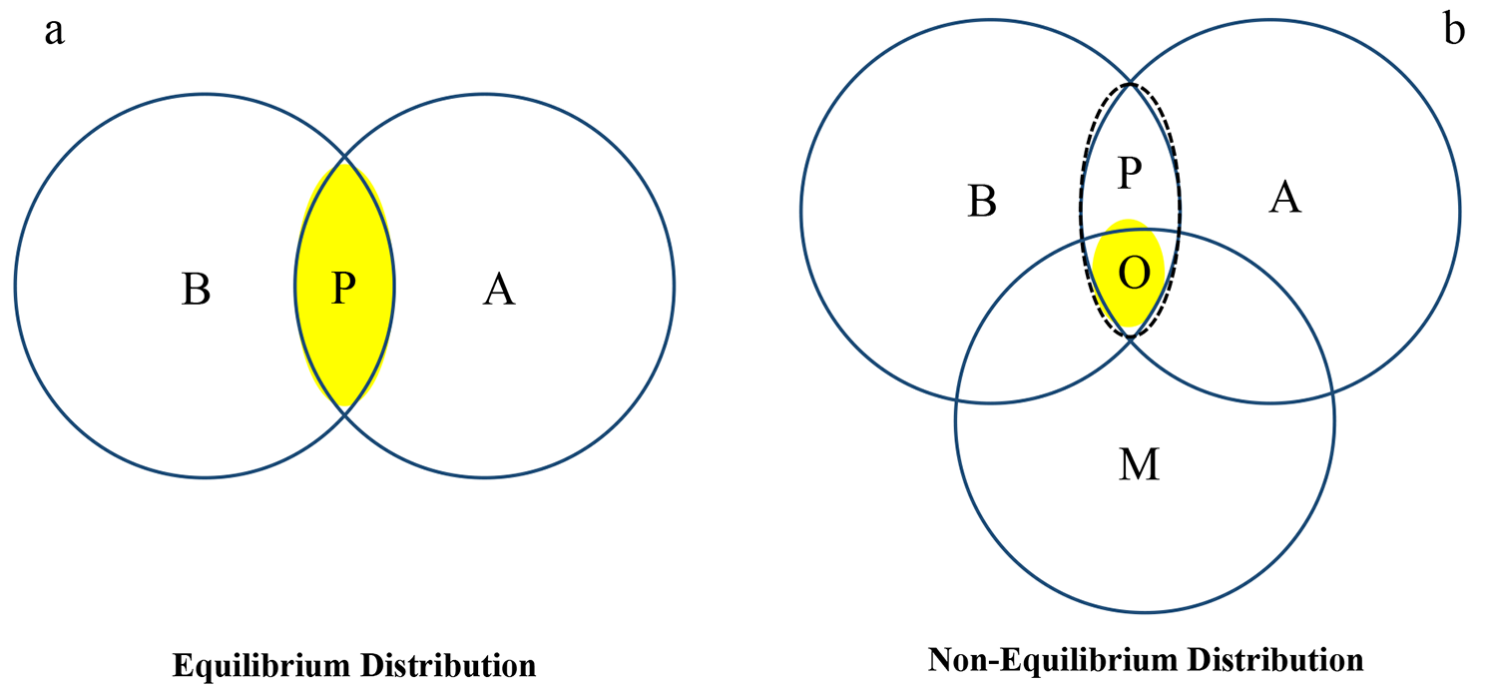
A comparison between equilibrium (panel **a**) and non-equilibrium (panel **a**) distributions and their influence on a species’ occupancy of its existing fundamental niche (circle **A**). In panel a, when a species exhibits an equilibrium distribution, it occupies all potentially suitable areas worldwide, resulting in a filled potential niche. Conversely, in panel b, if a species demonstrates a non-equilibrium distribution, its potential niche remains partially unoccupied due to the dynamic nature of areas over seasonal and long-term periods. In this figure, **A** represents abiotic, **B** represents biotic, P represents potential niche, M represents migration, and O represents occupied niche or realized niche

Researchers often overlook the transient nature of species distributions, if species have achieved equilibrium distributions where their current geographic ranges accurately reflect the balance between suitable biotic (Fig. 1- Circle B) and abiotic environments (Fig. 1- Circle A). However, it’s widely recognized that species distributions are typically in a non-equilibrium state (Fig. 1- panel b) in real-world scenarios, influenced by seasonal variations or migration (Fig. 1- circle M) [8, 9]. When a species’ range is not in equilibrium, its current distributions may not fully represent its entire range of physiological tolerances (Fig. 1- Circle A) [10]. Jackson and Overpeck [11] introduced the concept of the “potential niche,” (Fig. 1- oval P) referring to the part of the fundamental niche comprising all favorable abiotic (Fig. 1- circle A) and biotic (Fig. 1- circle B) conditions within a specific region and time. These biotic factors include the presence and abundance of mutualists, facilitators, predators, parasites, pathogens, and competitors, all shaping a species’ distribution [12].

Figure 1 illustrates comparisons between equilibrium (panel a) and non-equilibrium (panel b) distributions and their impact on a species’ occupancy of its existing fundamental niche (A = Abiotic). In this study, we define the fundamental niche as all suitable abiotic variables denoted by A. In panel a, when a species has an equilibrium distribution, it occupies all potentially suitable areas worldwide, filling its potential niche (P = Potential niche). Here, the potential niche can be considered as the “realized niche” in Hutchinson’s definition of niche. This potential niche represents the intersection of abiotic (Circle A) and biotic variables (Circle B). In this scenario, we anticipate the species to occupy all potential niche space as it is suitable and fully accessible to them. Contrastingly, in panel b, when a species exhibits a non-equilibrium distribution (meaning it has not accessed all parts of its potential niche, for example, due to migration barriers), its potential niche (P = dashed oval) remains partially unoccupied due to the dynamic nature of areas over seasonal and long-term periods. In panel b, M stands for migration, and in this panel, the realized niche would be part of the potential niche that is already occupied (Circle O) by the species. O stands for the occupied niche.

### Niche similarity vs. niche divergence

Niche conservatism (NC) [13], refers to the tendency of species to maintain characteristics of their fundamental niche over time. If fundamental niches remain unchanged, species are limited to colonizing regions with climates resembling those of their native range [7, 14, 15]. Additionally, if climate tolerances are conserved, species are expected to adjust their geographic ranges to match their historical climate conditions in response to global warming [16]. Species that cannot adapt or shift their ranges, due to factors such as area destruction or geographic barriers, may face extinction risks [17]. Furthermore, consistent parallels between the climate distributions of species in their native and non-native ranges are anticipated if NC in climate tolerance determines species’ range limits [13]. Consequently, if climate conditions in the non-native region resemble those in the native range, the species exhibits NC. Conversely, significant differences in climate conditions between native and introduced ranges suggest divergence in the ecological niche of the species in its non-native range [18].

Given the concept of climate niche conservatism (NC), the distribution of species in their native areas can provide insights into where they might successfully invade and subsequently expand their range [14, 15]. In the context of biogeographic hypotheses, a fundamental concept is that climateally unsuitable conditions can act as limiting factors for geographic ranges, especially when climate niche conservatism is at play. Identifying and testing such unsuitable conditions can be achieved through the use of species distribution models (SDMs) [19]. Warren et al. (2008) proposed that the detection of NC entails testing two hypotheses, namely niche equivalency (whether native and non-native niches are indistinguishable) and niche similarity (whether niches are more similar than expected by chance). Niche divergence refers to the alteration in the ecological niche (both fundamental and realized) resulting from constraints imposed by abiotic factors (such as climate) and biotic factors (like competition, predation, parasitism, etc.) on a subgroup of the population, followed by adaptation to this divergent niche [20]. These changes can ultimately contribute to the process of speciation [21].

### Climate and species distribution

Climate plays a crucial role in shaping the ecological niche of species. Organisms are finely attuned to their area, and any shifts in prevailing climate conditions can impact their typical behaviors. Factors such as temperature, precipitation, and solar radiation directly influence the feeding and mating habits of organisms. Terrestrial species typically thrive within specific climate conditions, referred to as their climate niche, encompassing temperature and precipitation ranges [20, 22].

Many studies have emphasized the significance of climate variables in shaping species distribution, particularly among mammals [23–31]. For example, for North American mammals, Billman, Beever [31] discovered that the best regression model involved five environmental variables, which encompassed seasonal temperature extremes, annual energy and moisture levels, and elevation. These variables collectively accounted for 88% of the variability in species density across the entire continent.

### Persian fallow deer

The fallow deer (*Dama mesopotamica*, Brook 1875) is a member of the Cervidae family [32]. It consists of two distinct species: the Persian Fallow deer (*Dama mesopotamica*) and the European Fallow deer (*Dama dama*) [33]. The Persian fallow deer once ranged from Iran and Iraq to Syria, Israel, and Palestine and inhabited certain southern regions of Turkey [34]. Meanwhile, the European fallow deer occupied the areas to the north and west of this region [33, 35, 36]. These species are now either extinct or endangered in their native ecosystems and are protected by their respective governments [37]. In under a century, the fallow deer (*Dama spp*.) has transitioned from the brink of extinction to establishing itself as one of the most successful hoofed animal species globally [38–40]. *Dama dama*, or the European fallow deer, is not facing significant global threats and is categorized as “least concern” on the IUCN list [39]. On the other hand, *D. mesopotamica*, the Persian fallow deer, remains at serious risk in its Turkish area and is safeguarded by the Turkish government [41]. However, the Persian fallow deer has flourished and adapted effectively in most regions, significantly contributing to food security and sustainability [39].

Until 1945, the Persian Fallow deer was believed to be extinct. In 1964, a small population of this deer was rediscovered in the Dez and Karkheh National Park. From as early as 1964, a program aimed at managing the species’ captive population had commenced [42, 43]. At that time, six deer were relocated from the Karkheh region to an enclosure in the Dasht-e-Naz region in Sari Township in Northern Iran. In subsequent years, in 2007 and 2008, approximately 70 deer were translocated from Ashk Island, Lake Urmia, and the Dasht-e-Naz region to two enclosures established in forests and woodlands close to the Dez and Karkheh Rivers [44]. According to Iran’s Department of Environment (www.doe.ir) annual wildlife census, the current population of the Persian fallow deer and the number of enclosures dedicated to conserving this species have increased significantly. Presently, more than 300 deer are living in a total of 12 enclosures that have been established throughout the country [43–45].

Reintroduction involves releasing a species into an area where it was once native but had subsequently gone extinct, a well-established and enduring practice [46]. The guidelines propose conducting preliminary studies to ascertain the specific area needs of the species, drawing insights from previous reintroduction initiatives involving similar species, assessing potential locations within the species’ historical range, selecting genetically diverse individuals, and evaluating the socio-economic considerations associated with the project [46–48]. The Persian fallow deer has not been reintroduced to its original areas in Iran but has been translocated from its native regions, Dez and Karkheh National Park, first to Dasht-e-Naz in 1964 and subsequently to other parts of Iran. Consequently, this species has been translocated to new areas. In terms of reintroduction, given that the species has previously inhabited these environments, there are fewer concerns regarding its chances of survival. However, when translocating the species to new areas, there must be substantial similarities, such as climate, between the native and new environments.

Considering niche conservatism (NC) in terms of climate tolerances, it would be advisable to translocate Persian fallow deer into areas that closely resemble their original environments, such as the Dez and Karkheh National Park. If climate tolerance is a determining factor for a species’ range limits, we would expect to observe consistent similarities in the climate distribution of the species in both their native and translocated areas. To successfully translocate the Persian Fallow deer, the first step is to quantify the species’ climate niche breadth within its original area, which, in this case, is the Dez and Karkheh National Park. Subsequently, it’s important to identify regions that closely resemble the original climate, providing a similar niche breadth for the Persian fallow deer. Niche breadth refers to the range of environmental conditions or resources that a species can effectively utilize within its ecological niche [49]. A broader niche breadth signifies a species’ capacity to thrive within a wider spectrum of conditions or resources [50].

It’s noteworthy that the reports from Iran’s Department of Environment (www.doe.ir) do not include such evaluations, and in all cases, the deer have been translocated to entirely new areas rather than reintroduced. What’s particularly interesting is that these new areas are spread across various locations in Iran (see methods section), and at first glance, it is evident that these areas differ from each other in terms of climate. As a result, in the current study, our goal is to undertake a comprehensive comparison of all these new deer areas with the original area, the Dez and Karkheh National Park, focusing on the similarity of the climate niche between them. We also plan to utilize extrapolation tools [51] To estimate more reliable or potentially climatically suitable regions for introducing Persian fallow deer.

## Materials and methods

### Study area

**Fig. 2:**
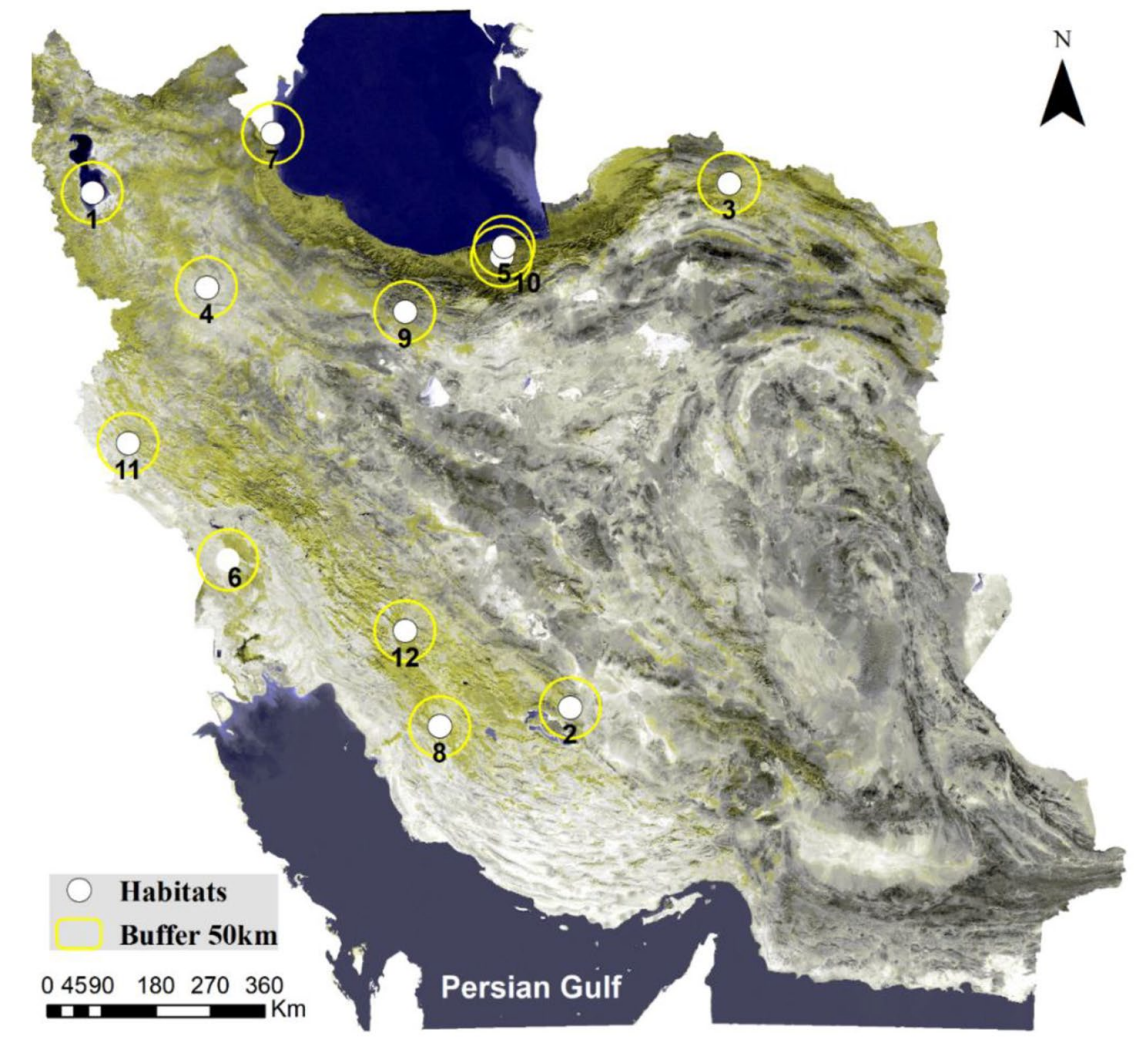
Study area location, 11 new area IDs, original area (ID = 6), and 50 km buffer around all areas

As previously mentioned, the Persian fallow deer have been translocated to 11 new areas, and a summary of these areas is provided in Table 1. In this table, the “ID” column indicates the identification number for each area, as depicted in Fig. 2, showing the location of new areas. The “Area” column lists the name of the region where the deer have been translocated. The “Area” column specifies the enclosed area’s size designated for this species. It’s worth noting that in nearly all instances, enclosed areas have been used for translocating the species. The “Buck,” “Doe,” and “Fawn” columns indicate the number of animals counted during the census for each respective year mentioned in the “Census” column. The “Origin” column specifies the source or origin of the translocated species, with a particular focus on the fact that most of the species originate from the Dasht-e-Naz Sari region. The “Number of Translocated” column records the count of individuals who have been translocated to the new areas, while the “Translocation Year” column indicates the year in which these translocations occurred. The Growth Rate” column displays the population growth rates in each area, taken from Goudarzi, Hemami [34]. It’s important to note that the information in this table may exhibit slight variations in different reports. However, the data presented here has been sourced from the official reports available from the Department of Environment (www.doe.ir).

**Table 1:** Summary of Persian fallow deer translocations to new areas

ID	Area	Area (Ha)	Buck	Doe	Fawn	Census date	Origin	Num. Translocated	Translocation Year	Growth Rate
1	Ashk Island	2150	90	152	64	2009	Dasht-e-Naz	6	1986	0.1
2	Bagh-e-Shadi	70	2	1	3	2010	Dasht-e-Naz	9	2005	0.04
3	Baba aman	-	6	5	2	2010	Pardisan	-	-	-
4	Bijar	14	2	2	4	2010	Ashk Island	6	2006	0.17
5	Dasht-e-Naz	55	16	7	5	2010	Dez and Karkheh	6	1964	0.18
6	Dez and karkheh	400	24	32	2	2010	Dasht-e-Naz	28	2007	-
7	Lavandevil	14	4	4	-	2010	Dasht-e-Naz	11	2006	-0.11
8	Miankotal	200	28	29	6	2010	Dasht-e-Naz	20	1993	0.06
9	Pardisan	-	2	3	1	2010	-	-	-	-
10	Semeskandeh	170	10	2	-	2010	Germany	7	1972	-0.26
11	Tunel-e-Reno	85	6	10	-	2010	Ashk Island	6	-	0.32
12	Tang-e Putak	15	9	4	4	2011	Dasht-e-Naz	15	2008	0.02

### Environmental variables

In new areas, Persian deer are confined to enclosed areas where predators are absent, and their populations are sustained by human feeding. Moreover, these species are insulated from factors such as farming and roads, which are identified as primary human pressures within Iran’s protected areas [52]. Consequently, only factors such as parasites, food quality, and, notably, climate variables dictate whether they can achieve growth rates in these areas. To assess the climatological similarity between original and new areas for Persian fallow deer, downloaded bioclimate layers as affecting factors on Persian fallow deer survival from the WorldClim database (www.worldclim.org*).* The Bioclimate data in the WorldClim database includes 11 temperature variables and 8 precipitation variables, with a spatial resolution of approximately 1 km. These climate data represent an average spanning the years between 1970 and 2000, encompassing a 30-year climate period. Given the proximity of these dates to the translocating of deer to new areas, they apply to the current study. These 19 variables often have a high correlation with each other, and therefore, it is recommended to use only some of these variables in species distribution modeling [16, 53, 54]. For this purpose, we used the usdm [55] package to exclude the highly correlated variables from the set through a stepwise procedure based on (The variance Inflation Factor) VIF. The remaining variables include Mean Diurnal Range (Bio 2), Temperature Seasonality (Bio 4), Mean Temperature of Wettest Quarter (Bio 8), Mean Temperature of Driest Quarter (Bio 9), Precipitation Seasonality (Bio15), Precipitation of Warmest Quarter (Bio 18), and Precipitation of Coldest Quarter (Bio 19).

### Calculating E-Space overlap, and equivalency

To evaluate the level of similarity in the environmental space between the original and new areas for Persian fallow deer, we calculated the ecological space within 15 and 50-km buffer zones surrounding the enclosed areas. This approach assumes that the climatic conditions within each enclosed area can be influenced by the conditions within a 15 km and 50 km buffer, offering two scenarios for assessing the climatic overlap between areas. To do this, we established buffers around each area listed in Table 1 and collected the climatic data within these buffers in ArcGIS software. Subsequently, we applied an ordination technique that utilizes kernel smoothers to analyze climate data within the environmental space [56]. Ordination is a method used to organize and understand data in a way that reveals patterns and relationships. It’s like arranging things neatly so you can see how they relate to each other. Principal Components (PCs) are the output of ordination techniques such as Principal Component Analysis (PCA) or Correspondence Analysis (CA). These techniques help to reduce the dimensionality of the data and reveal underlying patterns and relationships. PC1, and PC2, represent the main directions of variation in the data, with each PC capturing a different pattern of variation.

We divided the environmental space into a grid comprising 1000 × 1000 cells, where each cell represents a unique vector of available environmental conditions within the study areas. In this study, the environmental overlap between pairs of areas was calculated using Schoener’s D statistic [57]. The value of D varies between 0, indicating no overlap in the environmental space between the two areas, and 1, signifying that the two areas share the same environmental space. We employed the environmental equivalency test to evaluate whether the environmental spaces of pairs of areas are significantly distinct and whether the two environmental spaces are interchangeable. To conduct the environmental equivalency test, we compared the environmental overlap values (D) of pairs of areas to a null distribution of 100 overlap values. We inferred the equivalence of environmental spaces when the Schoener’s D overlap value of the areas being compared was not significantly different from the overlap values from the null distribution (*P* ≤ 0.05) [57]. The niche equivalency test is a one-tailed statistical test of the null hypothesis that niches are equal. This test compares the observed niche similarity (Schoener’s D statistic) between two areas with the overlap of niches generated from repeated resampling of occurrences from both areas. A null distribution is created from all iterations of niche similarity values obtained from the reshuffled occurrences. The observed niche similarity between the two areas is then compared against this null distribution. A significant result indicates that the two area datasets are not statistically equivalent, thereby rejecting the null hypothesis that the species’ niches are equivalent [8, 58].

Additionally, in the case of E-space analysis, the environmental overlap can be disentangled into three categories: Niche unfilling, niche stability, and niche expansion [59]. Unfilling typically represents the fraction of the ecological space of the original area that does not overlap with the new areas. Conversely, expansion denotes the portion of the ecological space of new areas that lacks overlap with the environmental space of the original area. Stability is the proportion of the ecological space of the original area overlapping with the environmental space of new areas [12]. All environmental space overlap analyses were conducted using R’s ‘ecospat’ package [59].

### Extrapolation modeling

Several valuable approaches have recently been proposed to detect and visually represent new environmental conditions based on native species’ areas [51, 60, 61]. New environmental conditions can be classified into two categories: (1) For a specific individual variable, the values may fall outside the range covered during training, referred to as univariate or strict extrapolation. (2) Certain areas in the environmental space may lie within the range of individual variables but constitute new combinations of predictors, known as multivariate or combinational extrapolation [62]. To manage this risk and identify analogous environments, we can employ three different approaches by comparing original and new areas: (a) Analysis of Multivariate Environmental Similarity Surface (MESS) [60], which provides a measure of how environmentally similar each location is to the median of the most dissimilar variable. (b) Mobility-oriented parity (MOP) [51] is a method that pinpoints areas of strict extrapolation and quantifies the environmental similarity between the calibrated and projected regions. (c) Extrapolation detection (ExDet) [61] is a technique that detects similarities or novel environmental conditions between native and invaded areas.

In this study, we employed MOP analysis because the MESS tool identifies extrapolation or ‘dissimilar’ points based solely on the ranges of individual (univariate) predictors. It does not consider the correlation structure, so it doesn’t account for new multivariate combinations of the covariates that might be included in the model [51]. Strict extrapolation occurs when the environmental conditions in the set of interest (new areas) are entirely outside the range of conditions observed in the reference set or calibration area (original area). When the MOP metric gives a value of 0 for a particular location (or grid cell) in the set of interest, it means that at least one of the environmental variables in that location is completely outside the range of values found in the reference set. This indicates that the area has novel environmental conditions that the model has never encountered before. Because the model lacks prior data on these new climate conditions, its predictions for these areas are highly uncertain and potentially unreliable, making these areas high-risk in terms of prediction reliability. Conversely, a MOP value of 1 signifies that the environmental conditions at a specific location in the set of interest are identical to those found in the reference set. This means that the model has encountered these exact conditions during its training phase. Because the model has seen these exact conditions before, it can make predictions with a higher degree of confidence. Therefore, predictions in these areas are more reliable, as they are based on known conditions from the training data [63, 64].

We used the NicheToolBox (ntbox) R package to perform this analysis [65]. The *mop* function in this package requires two types of raster stacks: (1) `M_stack`: a RasterStack (bioclimatic variables for reference set) containing variables representing the calibration area (original area). (2) `G_stack` is another RasterStack (bioclimatic variables for a set of interest) containing variables representing the areas or scenarios to which our extrapolation models will be transferred (the whole of Iran). A mobility-oriented parity RasterLayer where values of 0 represent strict extrapolation, which means complete dissimilarity of environments between the calibration (M_stack) and projection area (G_stack) [65]. We must account for a zone surrounding the areas to establish the calibration area. We opted for a 50 km buffer around each area and masked the bioclimatic variables in ArcGIS software to create an `M_stack` layer. To estimate more reliable or potentially climatically suitable regions for translocating Persian fallow deer, we examined four scenarios: (1) extrapolation using the original area, Dez and Karkheh National Park. (2) extrapolation relying exclusively on Dasht-e-Naz. (3) extrapolation based on Ashk Island, and (4) a combined approach involving all three areas.

## Results

### E-space niche overlap

**Fig. 3:**
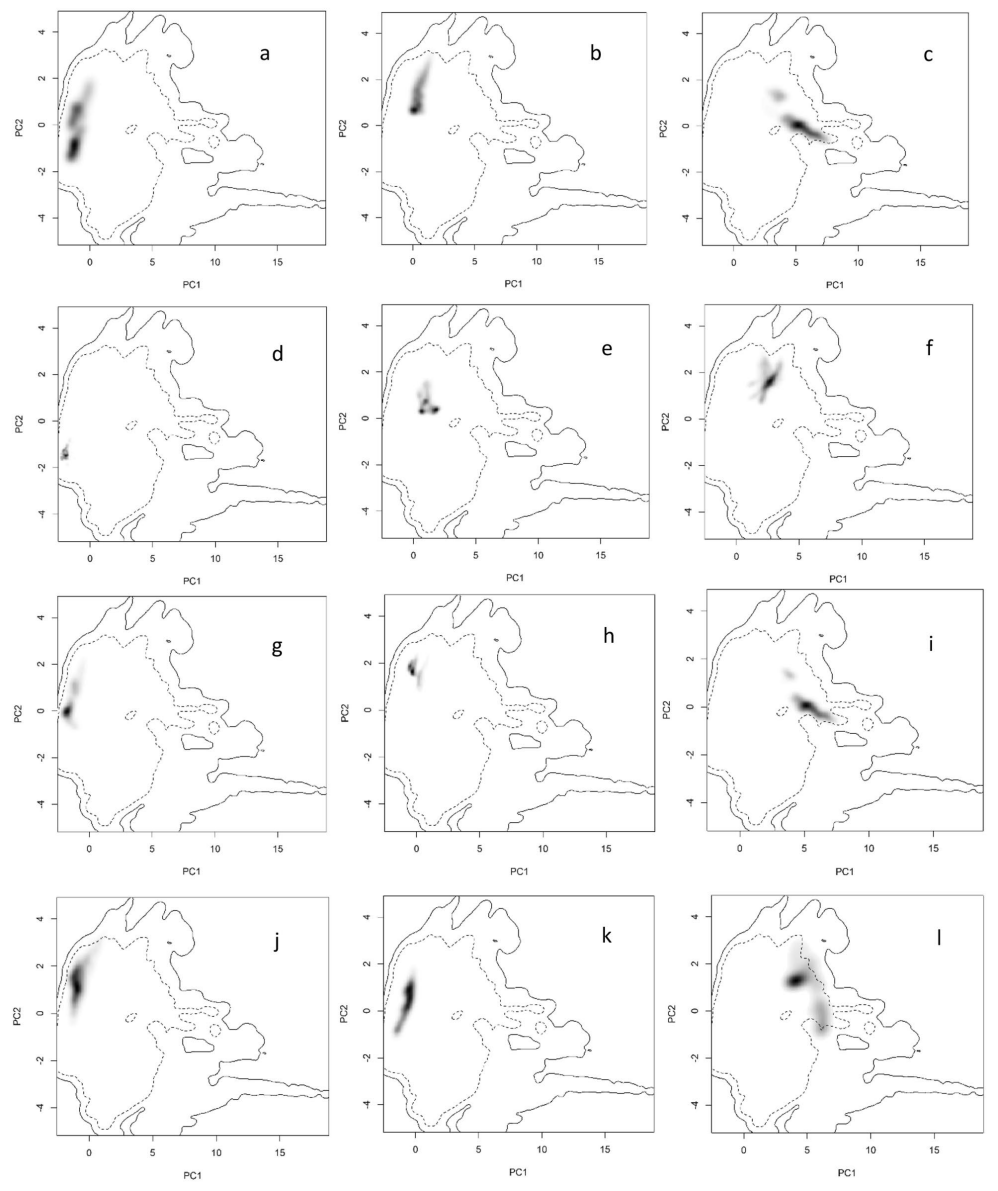
Niche breadth of the original area (Dez and Karkheh) and 11 new areas in environmental space produced by the principal component analysis method (PCA-ent). The grey-to-black shading represents the grid cell density of the climatic values (black being the highest density). The first dashed line represents 50% of the available environment and the solid line represents 100%. (**a**) Miankotal, (**b**) Pardisan, (**c**) Semeskandeh, (**d**) Dez and Karkheh, (**e**) Ashk Island, (**f**) Baba aman, (**g**), Bagh-e-Shadi (**h**) Bijar, (**i**) Dasht-e-Naz, (**j**) Tang-e Putak, (**k**) Tunel-e-Reno, (**l**) Lavandevil

Figure 3 illustrates the E-space plots of the 12 analyzed areas for Persian fallow deer within the environmental space generated by the principal component analysis (PCA-ent) method. The PCA-ent results depict each area E-space in the two primary axes relative to the environmental conditions across the entire study area. The shading, ranging from grey to black, represents the grid cell density, with black indicating the highest density of climatic variables at a 50 km buffer around all areas. The first dashed line signifies 50% of the available environment, while the solid line denotes 100% (whole Iran). In the context of E-space plots and environmental analysis, “density” refers to the concentration or frequency of grid cells within the plotted environmental space that share similar climatic conditions. This concept is visually represented by the shading of grid cells on the plots, where darker shades (often black) indicate areas with a higher density of grid cells exhibiting similar climatic variables.

Figure 3-e shows the Persian fallow deer’s origin area, including Dez and Karkheh National Park. Although we have expanded our assessment to include a radius of up to 50 km around this area to examine area similarity, it is evident that the climatic variations in this region are minimal. A brief observation of Fig. 3 reveals that none of the 11 new areas for Persian fallow deer in Iran coincide with the original area in terms of climate. This signifies that the currently translocated deer individuals are experiencing significantly different climatic conditions than their native area.

### Niche equivalency

In all conceivable pairwise comparisons among origin and new areas, we observed the rejection of the null hypothesis in the niche equivalency test (Table 2). According to Table 2, the degree of overlap (Schoener’s D) between the original area and the new areas is nearly zero across all cases. These statistics indicate that new areas are fundamentally distinct from the original area regarding climate. Furthermore, when examining niche expansion, unfilling, and stability, it becomes evident that the disparities between their climatic niches are highly maximum. The outcomes of the niche expansion, unfilling, and stability assessments reinforce the notion that Persian fallow deer has diverged its climatic niche in Iran.

**Fig. 4:**
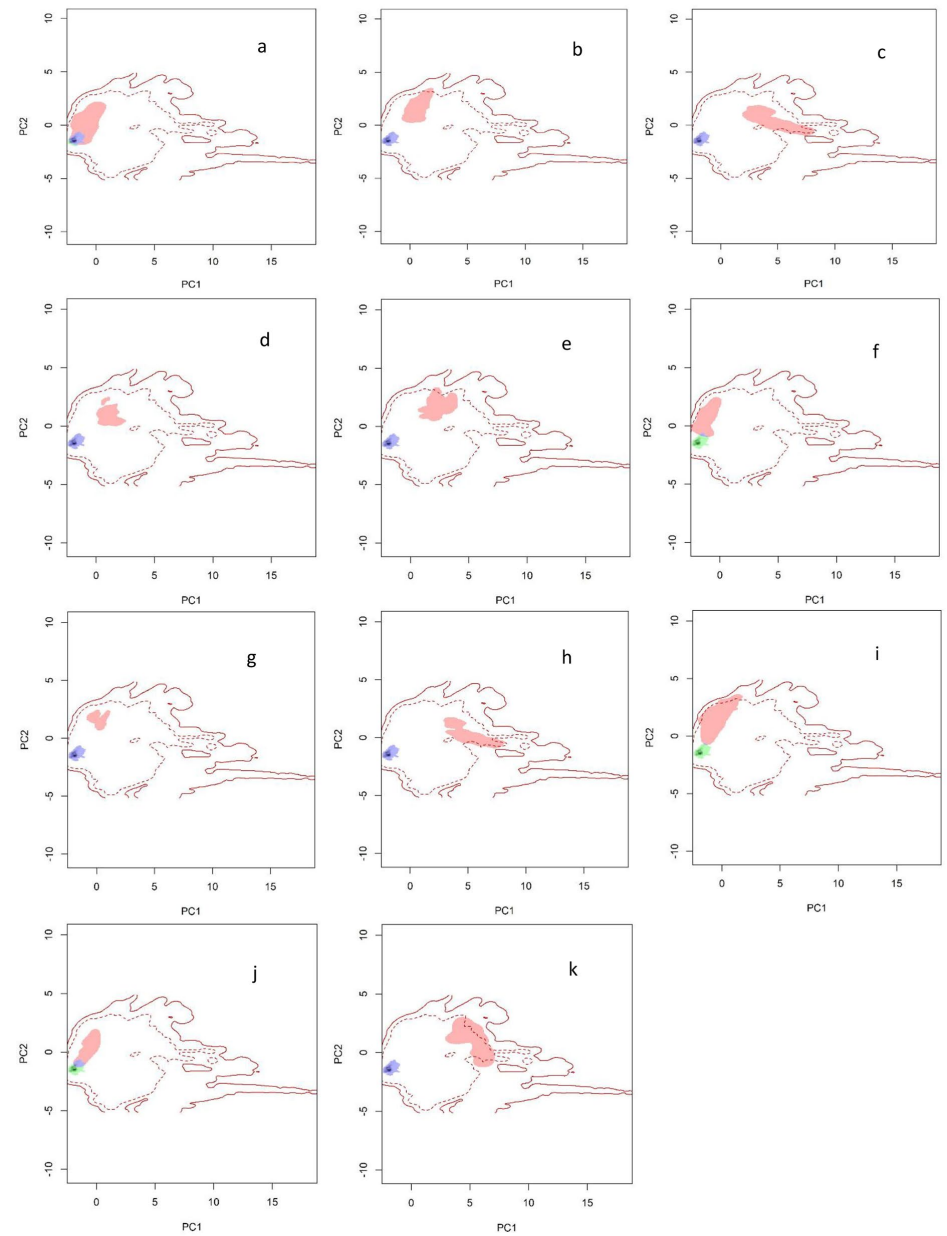
Climate niche surfaces overlaid between the original habitat (Dez and Karkheh) and 11 new habitats. The pair of surfaces for each habitat was created on one of three background extents that gave the best environmental niche model transferability. The first two axes from the underlying principal components analysis are shown on the x-axis (PC1) and y-axis (PC2). Within each plot, the green area indicates niche unfilling, blue indicates niche stability, and red indicates niche expansion. The solid lines indicate 100% (Iran) of available climates for each background, and the dashed line represents 50% of available climates. (**a**) Miankotal, (**b**) Pardisan, (**c**) Semeskandeh, (**d**), Ashk Island (**e**) Baba aman, (**f**), Bagh-e-Shadi (**g**) Bijar, (**h**) Dasht-e-Naz, (**i**) Tang-e Putak, (**j**) Tunel-e-Reno, (**k**) Lavandevil

Figure 4. Also shows the overlapping climate niche Surfaces between the original area and the new areas. Each area pair of surfaces was constructed using one of three background extents that offered the highest transferability for environmental niche modeling. The x-axis (PC1) and y-axis (PC2) display the first two axes derived from the principal components analysis. In each plot, the green region represents niche unfilling, blue signifies niche stability, and red denotes niche expansion. The solid lines depict 100% of the available climates (Iran) for each corresponding background, while the dashed line indicates 50% of the available climates. In this figure, the original area of Dez and Karkheh National Park is depicted in blue. At the same time, each red region represents the new areas’ full climatic range. Like Fig. 3, this one also reveals that niche expansion has occurred across all new areas. Nevertheless, there is minimal overlap in the two cases.

**Table 2:** E-space comparisons for persian fallow deer areas in Iran

	Overlap		Niche equivalency		
Pairs	D	Expansion	Stability	Unfilling	*P*-value
Original vs. Ashk Island	0.07	0.75	0.25	0.05	0.01
Original vs. Bagh-e-Shadi	0	1.00	0.00	1.00	0.01
Original vs. Baba aman	0	1.00	0.00	1.00	0.01
Original vs. Bijar	0	1.00	0.00	1.00	0.01
Original vs. Dasht-e-Naz	0	1.00	0.00	1.00	0.01
Original vs. Lavandevil	0	0.99	0.01	0.99	0.01
Original vs. Miankotal	0	1.00	0.00	1.00	0.01
Original vs. Pardisan	0	1.00	0.00	1.00	0.01
Original vs. Semeskandeh	0	1.00	0.00	1.00	0.01
Original vs. Tunel-e-Reno	0.02	0.93	0.07	0.88	0.01
Original vs. Tang-e Putak	0	1.00	0.00	1.00	0.01

### Extrapolation analysis

**Fig. 5:**
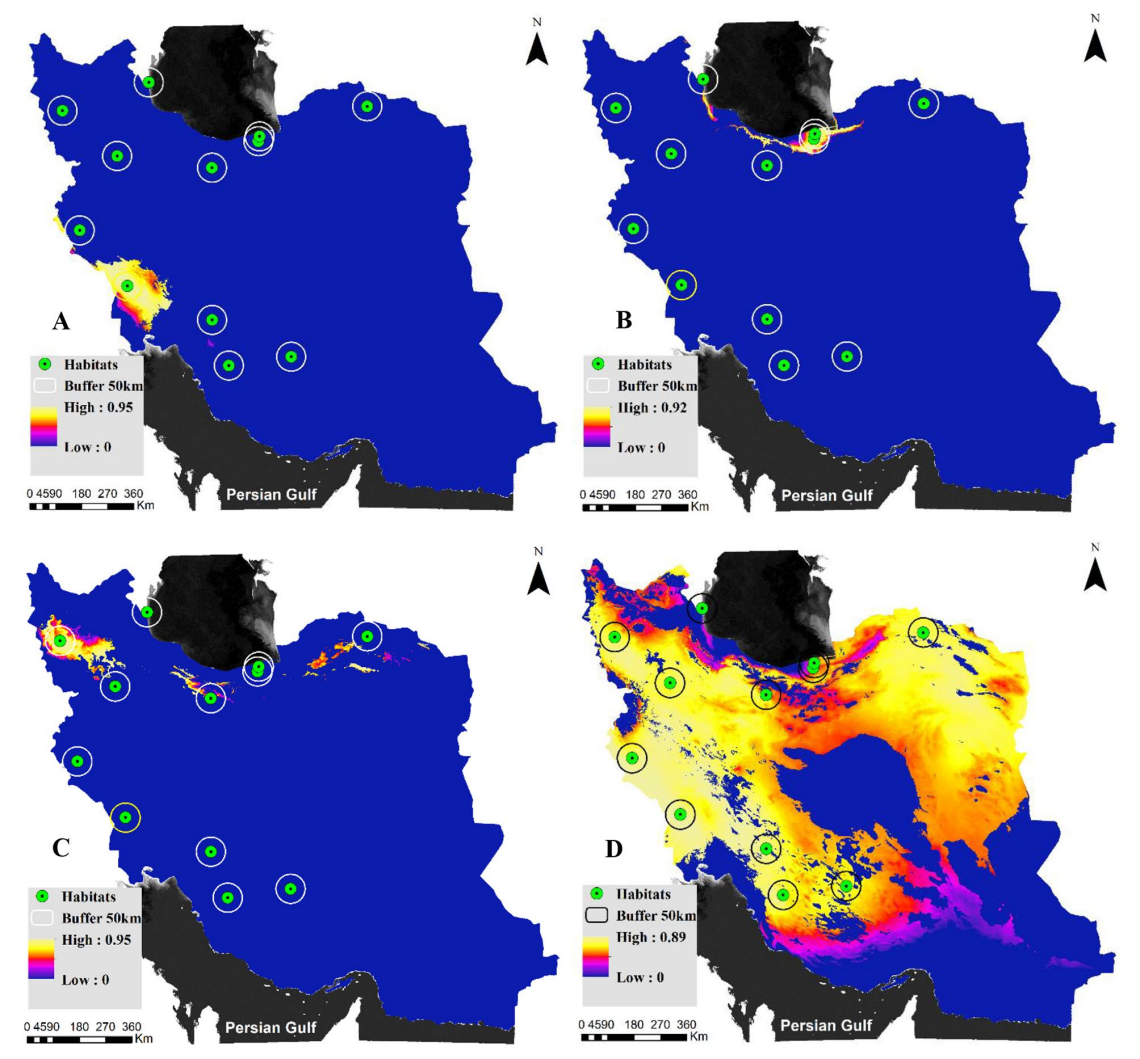
Extrapolation maps of Persian fallow deer in Iran. (**A**) based on the original, (**B**) based on Dasht-e-Naz, (**C**) based on Ashk Island, (**D**) based on the original, Dasht-e-Naz, and Ashk Island areas

Figure 5 illustrates extrapolation maps for Persian fallow deer in Iran. As previously mentioned, mobility-oriented-parity (MOP) analysis facilitates the comparison of environmental conditions between a reference set and a set of interests. The primary objectives of the MOP analysis are to identify non-analogous conditions in the set of interest compared to the reference set and to quantitatively assess the degree of dissimilarity in these conditions. These analyses explore potential habitats where deer could be successfully translocated based on their original habitat preferences. Decisions based on predictions from these areas can be made with greater confidence due to robust data support from the model. However, areas with low MOP values do not necessarily indicate unsuitability; rather, translocations to these areas should proceed cautiously due to the higher uncertainty involved. Figure 5-A suggests that optimal translocation sites can be predominantly near Dez and Karkheh National Park. Conversely, Fig. 5-B presents the extrapolation map centered on Dasht-e-Naz, a historically significant area for Persian fallow deer populations. This map shows only limited areas as highly suitable with confidence, and none of the proposed new habitats align with these criteria, except for the Semeskandeh region.

Ashk Island (Fig. 5-C) within Urmia National Park has proven successful in translocating Persian fallow deer. Extrapolation from this region indicates limited similar climatic areas for the species across Iran. Considering the climatic suitability observed in the original, Dasht-e-Naz, and Ashk Island for this species, we conducted extrapolations across all three areas. The result (Fig. 5-D) is a map outlining regions in Iran that could potentially support the translocation of fallow deer under current conditions. This map highlights that a substantial portion of Iran’s climate aligns closely with these three mentioned areas.

## Discussion

Our analysis of climate niche similarity of Persian fallow deer areas in Iran reveals significant differences in climate conditions between the original area (Dez and Karkheh) and the 11 new areas. These differences indicate that the translocated deer are exposed to markedly distinct climates compared to their primary environment. Niche equivalency tests confirmed the substantial dissimilarity, with almost no overlap in climate niches between the original and new areas, underscoring the fundamental contrast in climate conditions.

The situation regarding deer in Iran has unfolded in a way where Iran’s Department of Environment, over the past five decades, has translocated this species without conducting expert investigations into new areas to safeguard it from the risk of extinction. While the Persian fallow deer now maintains a stable population in Iran and the most of new areas, the growth rate is positive [34], it’s crucial to acknowledge that previous attempts to translocate this species to various other regions have not been successful [43]. However, setting aside any potential bias associated with the activities of the Department of Environment in Iran, it is presently reported that more than 400 deer are inhabiting a total of 12 enclosures established across the country [43–45]. While translocation to enclosed areas appears to have been successful in terms of increasing the species’ population under human care, it’s important to note that translocation success is ultimately defined as the establishment of self-sustaining new populations in the target areas [66] for a significant duration, contingent upon the lifespan of the species [67]. To assess the effectiveness of translocation programs, various criteria have been reported, including the establishment of a Minimum Viable Population (MVP), successful breeding of the first wild-born animals, achieving a positive recruitment rate over three years, quantifying post-release survival and reproduction rates, and determining the finite rate of increase [34, 67, 68].

While Persian deer have indeed been translocated into enclosed areas under human care, rather than released into natural environments where factors such as predator presence, food availability, vehicle collisions, hunting, and disease could affect their population, it is essential to recognize that determining the true success of translocation extends beyond the scope of the present study. Our primary goal was to assess the climate niche similarity between the original area and the new enclosed areas, rather than evaluating the overall success of the translocation effort in terms of establishing self-sustaining populations in the target areas. Hence, given the absence of climate overlap between the original and new areas, yet observing a growth rate for this species, it appears that the species in Iran exhibits a non-equilibrium distribution and may not have accessed other parts of the country. Therefore, it is plausible that new areas with a growth rate could be considered as potential niches for this species. Consequently, we cannot attribute the increased population of this species to niche divergence and speciation. It’s worth noting that this species, or its closely related European counterparts, has been translocated to various regions worldwide, including Britain [69, 70], Israel [71], Europe [72, 73], Japan, Australia, New Zealand, and South America [73]. An extensive evaluation of the status of this species outside of Iran is beyond the scope of our study. However, brief assessments suggest the success of these translocations in other parts of the world [73].

Our extrapolation analysis demonstrated that, based on the climate conditions of the three areas (Dez and Karkheh, Dasht-e-Naz, and Ashk Island), nearly all the new areas for this species seem to be climatically suitable. It’s worth mentioning that our analysis considered a 50 km buffer, and if this buffer is reduced, the areas deemed suitable for deer from a climate perspective may decrease. We found that translocating Persian deer in areas primarily resembling the original area, was best suited around Dez and Karkheh National Park. Other regions, such as Dasht-e-Naz and Ashk Island, have also historically supported Persian fallow deer populations. While these areas presented some limited climatically similar regions for translocation, they did not align with the designated new area, except for the Semeskandeh region in the case of Dasht-e-Naz. Despite variations in the suitability of different regions, the extrapolation maps collectively indicated that a substantial portion of Iran’s environmental conditions fall inside the range observed in the three reference sets. If we solely rely on predictions from the Dez and Karkheh area, the reliability of model predictions for translocating Persian fallow deer may be reduced. It is crucial to exercise caution and make informed decisions when using these predictions. These findings may support the potential success of deer translocation in Iran, while also highlighting the need for careful consideration of climate factors in area selection and species management.

However, in other regions where translocations have recently taken place, time is needed to ascertain whether the species can be established successfully. For instance, in the Miankotal region, where approximately 20 deer were translocated from Dasht-e-Naz in 1993, there are currently 63 deer, which is fewer than anticipated based on the Department of Environment’s reports. In the Bagh-e-Shadi region, only six out of the nine initially translocated deer have survived, and in the Lavandevil region, eight out of the 11 translocated deer are still alive [45]. It’s worth noting that the Department of Environment is the primary source of this information, published in a Persian report that requires updating. Consequently, it becomes challenging to gain a precise understanding of the deer’s status in each of the translocated areas.

## Conclusion

Our assessment of climate niche similarity between Persian fallow deer areas in Iran revealed marked disparities in climate conditions between the original area and the 11 new areas. As previously noted, asserting that Persian deer have successfully adapted to new areas is challenging due to their confinement and the removal of many population-limiting factors in enclosed areas by humans. However, we cannot overlook the fact that the population of this species has increased during the last five decades of preserving this species in Iran. Even if we mitigate all population-limiting factors in enclosed areas to facilitate maximum population growth, climate variables such as minimum or maximum temperature, which are not controlled by humans, may ultimately constrain the population growth of this species. Therefore, our most plausible assumption is that this species exhibited a non-equilibrium distribution in Iran and was hindered from accessing all suitable area parts across the country due to various obstacles. It is conceivable that numerous regions in Iran could potentially serve as suitable niches for this species, yet it has only managed to occupy a fraction of its potential area. The extinction of this species in countries like Iraq and Turkey further suggests that it had a broader distribution in the past, implying that its potential niche may indeed be extensive.

The management history of deer in Iran spans five decades of persistent efforts by the Department of Environment to translocate and conserve this species, despite encountering certain challenges. While the Persian fallow deer now maintains a stable population within enclosed areas, previous attempts to translocate the species in diverse regions have yielded opposite results. Newly translocated species require additional time for evaluation, with varying degrees of success observed. Furthermore, the species’ history of successful translocation in different parts of the world underscores its potential for thriving beyond the borders of Iran. To date, the organization has boosted the species’ population through rigorous protection and enclosure of their areas. However, the lack of expert translocations to new areas that account for climate similarities may result in population losses. Therefore, it is advisable to include climate considerations when translocating this species to new areas in Iran.

## Data Availability

Availability of data and materialNiche equivalency test details are available at https://github.com/ehsanrahimi666/Persian-Deer.git.
